# Characterization of *Shigella* virulence factor intracellular spread A (IcsA, or VirG) functional epitopes against *S. flexneri* 2a and *S. sonnei* invasion and adherence

**DOI:** 10.1128/aem.01175-25

**Published:** 2025-08-22

**Authors:** Aashwina Madhwal, Sai Simha Reddy Vakamalla, Siqi Li, Weiping Zhang

**Affiliations:** 1 Department of Pathobiology, University of Illinois at Urbana-Champaign 612102, Urbana, Illinois, USA; Centers for Disease Control and Prevention, Atlanta, Georgia, USA

**Keywords:** IcsA (VirG), *Shigella*, functional epitopes, vaccine, biogenesis

## Abstract

There are no licensed vaccines against *Shigella*, a leading cause of children’s diarrhea and travelers’ diarrhea. To develop a cross-protective vaccine against heterogeneous *Shigella* species and serotypes, we attempted to apply an epitope- and structure-based vaccinology platform, multiepitope fusion antigen, to construct an optimal polyvalent chimeric immunogen with functional epitopes from the key *Shigella* virulence determinants. With *Shigella* invasion plasmid antigens B and D functional epitopes identified in recent studies, in this study, we focused on *Shigella* intracellular spread protein A (IcsA; also known as virulence gene G, VirG), a multifunctional virulence factor that plays roles in bacterial adherence, invasion, and particularly intracellular and intercellular spread. We *in silico* predicted continuous B-cell immunodominant epitopes from the IcsA functional passenger domain, and we fused each epitope to a non-homologous carrier protein, *Escherichia coli* adhesin CS4 subunit CsaB, for epitope fusions. We then immunized mice with each epitope fusion and examined antibody functions against *Shigella* bacterial invasion and adherence. Eleven IcsA B-cell epitopes were identified and fused to CsaB. Mice intramuscularly immunized with each epitope fusion developed IgG responses. Mouse serum antibodies significantly reduced *Shigella sonnei* or *Shigella flexneri* 2a bacterial invasion or adherence *in vitro*, particularly from the fusion with epitope #2 (_122_GDNNDGNSCGGNG_134_), #3 (_156_GGSGADHNGDGGE_168_), #7 (_309_YIISGKEDDGTQNV_325_), #9 (_485_WNDTDGDSHG_494_), or #10 (_507_TILADNLSHHNIN_523_). These IcsA epitopes are more effective in inducing functional antibodies, suggesting their antigenic utility in constructing an optimal polyvalent *Shigella* immunogen and developing a cross-protective vaccine, and perhaps in better understanding their role in IcsA biogenesis.

**IMPORTANCE:**

An effective *Shigella* vaccine is urgently needed to reduce *Shigella*-associated diarrhea and dysentery. *Shigella* IcsA (or VirG) plays a crucial role in *Shigella* bacterial pathogenesis and is conserved across *Shigella* species and serotypes, thus making IcsA an excellent antigen target in *Shigella* vaccine development. Identification of the functional epitopes of the IcsA passenger domain from this study enables us to construct an optimal epitope- and structure-based polyvalent immunogen for a cross-protective multivalent *Shigella* vaccine. Unlike the lipopolysaccharide-based whole-cell or subunit vaccine candidates that are serotype-specific, a multivalent vaccine carrying functional epitopes of conserved virulence determinants would be cross-protective against the heterogeneous *Shigella* species and serotypes, effectively preventing shigellosis and dysentery. Data from this study may also provide insightful information for a better understanding of IcsA biogenesis.

## INTRODUCTION

*Shigella* is the most common bacterial cause of diarrhea in children younger than 5 years (1) and a major cause of epidemic dysentery (2, 3). Currently, no effective countermeasures, including licensed vaccines, are available against the largely antibiotic-resistant *Shigella* bacteria. Developing vaccines against *Shigella* has encountered various challenges historically. One key challenge is virulence and immunological heterogeneity among *Shigella* strains (2, 4). *Shigella* is a genus of four species, *S. flexneri*, *S. sonnei*, *S. boydii*, and *S. dysenteriae*, but over 50 serotypes based on their O-specific polysaccharides (OSP) of lipopolysaccharides (LPS) (5). An effective *Shigella* vaccine would need to protect the prevalent and the most virulent species and serotypes at least (2, 4).

A few cellular and acellular vaccine candidates have been investigated preclinically or clinically. Although they were demonstrated to be immunogenic and protective preclinically, or even well tolerated in humans, live attenuated or killed cellular products derived from *S. dysenteriae* type 1 (6, 7), *S. flexneri* 2a (8–11), or *S. sonnei* (12–16) are expected to be protective against homologous strains, but not cross-protective against the heterogeneous species and serotypes. Similarly, the acellular candidates, particularly those based on the LPS O-antigen of an individual serotype, such as OSP conjugates (17–23) or outer membrane vesicles (24–26), are also serotype-specific. On the other hand, protein-based acellular candidates derived from virulence determinants conserved across *Shigella* species and serotypes, including invasion plasmid antigens (Ipa) and intracellular spread protein A (IcsA, also known as virulence gene G; VirG will be used hereafter) (27–32) have shown promise in developing cross-protective *Shigella* vaccines, though a protein-based *Shigella* vaccine is yet to be achieved.

Differing from the above protein-based *Shigella* vaccine candidates that use one or two conserved virulence determinants as the antigen, we recently developed a multivalent *Shigella* vaccine by using a polyvalent chimeric protein immunogen to target IpaB, IpaD, VirG, and Shiga toxins (33). By applying a novel epitope- and structure-based vaccinology platform, multiepitope fusion antigen (MEFA) (34–36), we integrated the *in silico*-predicted immunodominant B-cell epitopes from IpaB, VirG, GuaB, Stx1A, Stx2A, and StxB into an IpaD backbone to mimic native antigenicity and construct a polyvalent *Shigella* MEFA protein. This *Shigella* MEFA was demonstrated to be strongly immunogenic, induced functional antibodies against *in vitro* invasion of *S. sonnei*, *S. flexneri* 2a, 3a, 6, *S. boydii*, and *S. dysenteriae*, and further protected against lethal pulmonary infection from *S. sonnei* and *S. flexneri* 2a, 3a, and 6 in a murine model (33).

Knowing that the immunodominant B-cell epitopes may not necessarily be functional or protective and that functional epitopes can only be identified empirically (37), we have reasons to believe that the epitopes included in the current *Shigella* MEFA may not all be functional. While this polyvalent protein induced protective antibodies against *Shigella* invasion and Shiga toxin cytotoxicity, the protection against bacterial invasion can be from the epitopes of IpaB, IpaD, and VirG collectively, the same as the epitopes of the A and B subunits of Shiga toxins (Stx and Stx2) against cytotoxicity (33). Additionally, even if individual epitopes in the current *Shigella* MEFA are functional, they may not be the top representative. Therefore, the current *Shigella* MEFA immunogen can be improved by including the better functional epitopes from each target virulence determinant. We have mapped the B-cell epitopes of IpaB and IpaD and empirically identified the top-ranked functional epitopes (38, 39). Still, we need to identify the functional epitopes for the Shiga toxins and VirG.

*Shigella* VirG encodes a self-associating autotransporter protein and comprises three segments: an atypical N-terminal signal peptide, a central passenger (or alpha, α) domain, and a C-terminal β barrel domain. The signal peptide containing the first 52 residues (residues 1–52) is cleaved off from the protein after transportation across the inner membrane and translocation to the periplasm. The β-barrel translocation domain, comprising residues 759–1102, inserts into and anchors at the outer membrane at the old pole of the bacterial cell and exports the passenger domain to the surface of *Shigella* bacteria (40–42). The surface-exposed VirG passenger domain (residues 53–758; ~75 kDa), including a subregion of glycine-rich repeats (GRRs; residues 117–332), is considered a member of the adhesin involved in diffuse adherence (AIDA) family and mediates *Shigella* bacterial adherence to host receptors before invading into host colonic epithelial cells (43, 44). After entering host cells, this VirG passenger domain recruits host actin polymerization regulator neural Wiskott-Aldrich syndrome protein (N-WASP) to form unipolar comet-like actin tails for actin-based motility (ABM), propelling *Shigella* bacterial intracellular movement and intercellular spreading (45–51). Thus, the passenger domain confers VirG multifunction: as the adhesin to bind host cell receptors (and to enhance bacterial invasion as an invasin [43]) and as an effector to spread *Shigella* bacteria in host cells intracellularly and intercellularly; thus, it is essential in *Shigella* pathogenesis (45, 52, 53). Consequently, VirG is targeted as an antigen for vaccine development (2, 54).

In this study, we attempted to identify the functional epitopes of the VirG passenger domain. We first predicted the continuous conformational B-cell immunodominant epitopes with online B-cell epitope prediction software. We selected the epitopes that scored at the top in terms of antigenicity and then fused each epitope to a carrier protein. Next, we immunized mice with each epitope fusion protein to evaluate immunogenicity specific to VirG. Finally, we examined the antibodies derived from each epitope fusion for functional activities against *Shigella* bacterial invasion and adherence to identify the functional VirG epitopes for future construction of an optimal *Shigella* MEFA protein antigen and development of a cross-protective *Shigella* vaccine.

## RESULTS

### *Shigella* VirG passenger domain epitopes and epitope fusions

**Fig 1:**
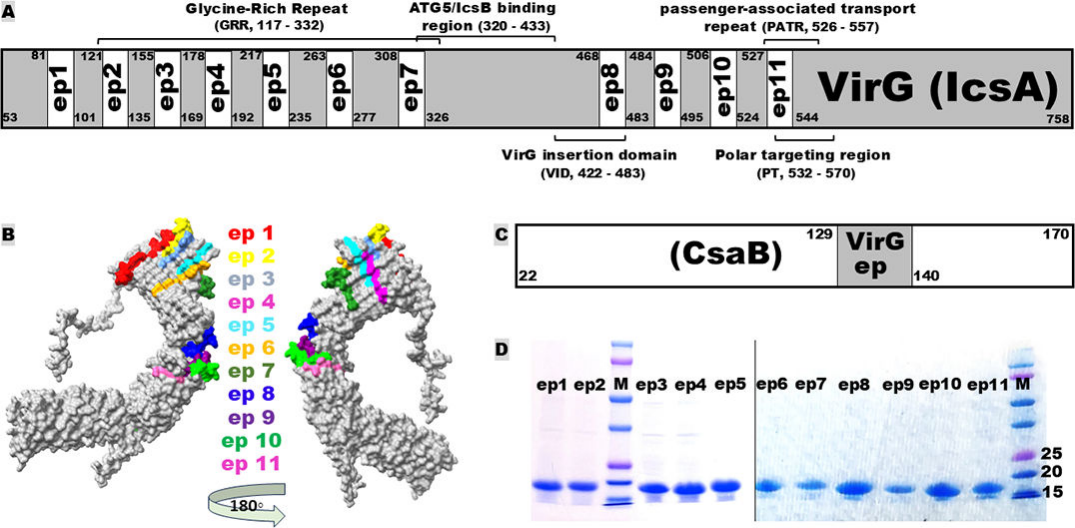
Schematic illustration of *Shigella* VirG (IcsA) passenger domain epitopes and epitope fusions. (**A**) A diagram shows the B-cell immunodominant epitopes on the VirG (IscA) passenger domain, with residue numbers outside each epitope and major functional regions marked (VirG functional domains are based on reference 55). (**B**) A VirG protein model (model ID AAA26547) shows the passenger domain epitopes (in different colors). (**C**) A diagram to show the insertion of a VirG epitope into carrier CsaB (the major structural subunit of enterotoxigenic *E. coli* adhesin CS4) for an epitope fusion. (**D**) SDS-PAGE shows the purified recombinant epitope fusion protein with Coomassie blue staining, molecular marker in kDa.

Eleven continuous B-cell immunodominant epitopes were identified from the passenger domain of VirG based on antigenic scores (Fig. 1). They are EP1 (_82_GAGEDGMDAWYITSSNPSH_100_), EP2 (_122_GDNNDGNSCGGNG_134_), EP3 (_156_GGSGADHNGDGGE_168_), EP4 (_179_NGEIISGGHGGDS_191_), EP5 (_218_SGGNGGNNYGEGDGGNG_234_), EP6 (_264_YDGYGGNAITGDN_276_), EP7 (_309_YIISGKEDDGTQNV_325_), EP8 (_469_TKSYISDQNKLIYG_482_), EP9 (_485_WNDTDGDSHG_494_), EP10 (_507_STILADNLSHHNIN_523_), and EP11 (_528_TKSGEGTLILAEKNTY_543_). Among them, EP2, EP3, EP4, EP5, EP6, EP7, EP8, and EP11 resided in the known functional or biogenic domains of VirG (Fig. 1A). All epitopes are 100% homologous among the *Shigella* species (*S. sonnei*, *S. flexneri*, *S. dysenteriae*, and *S. boydii*) and serotypes (*S. flexneri* 2a, 3a, and 6) we have examined. They are hydrophobic and surface-exposed on a VirG protein model (ID AAA26547) (Fig. 1B). Each epitope was then fused to a non-homologous protein carrier, CsaB, the major structural subunit of enterotoxigenic *Escherichia coli* fimbrial adhesin CS4. Epitope fusions were generated by substituting a nucleotide segment coding CsaB epitope (residues 130–139) with each VirG epitope sequence (Fig. 1C), using splicing overlap extension PCRs with specific primers (Table 1). Eleven VirG epitope fusion genes were constructed and expressed by *E. coli* strain BL21 for epitope fusion recombinant proteins (Fig. 1D).

**TABLE 1:** PCR primers used to construct VirG epitope fusion genes and amplify VirG gene segments^*a*^

Primer designation	Primer sequence	Purpose
ep1F	ATGGATGCGTGGTATATAACTTCTTCCAACCCCTCTCATttagtgattggtgcgactac	Paired w/ CsaB-R to insert ep12 into CsaB
ep1R	GTTATATACCACGCATCCATTCCATCTTCACCAGCTCCtgacgttccaaaatttaatt	Paired w/ CsaB-F to insert ep12 into CsaB
ep2F	AATGATGGTAATAGTTGTGGCGGTAATGGTttagtgattggtgcgacta	Paired w/ CsaB-R to insert ep2 into CsaB
ep2R	GCCACAACTATTACCATCATTATTATCACCtgacgttccaaaatttaatt	Paired w/ CsaB-F to insert ep2 into CsaB
ep3F	GGTGCTGACCATAACGGTGATGGTGGTGAGttagtgattggtgcgactac	Paired w/ CsaB-R to insert ep3 into CsaB
ep3R	TCACCGTTATGGTCAGCACCGCTACCGCCtgacgttccaaaatttaatt	Paired w/ CsaB-F to insert ep3 into CsaB
ep4F	AATTATTTCAGGTGGACATGGTGGCGATAGTttagtgattggtgcgactac	Paired w/ CsaB-R to insert ep4 into CsaB
ep4R	CATGTCCACCTGAAATAATTTCTCCATTtgacgttccaaaatttaat	Paired w/ CsaB-F to insert ep4 into CsaB
ep5F	GGTAACAATTATGGTGAGGGTGATGGCGGTAATGGAttagtgattggtgcgact	Paired w/ CsaB-R to insert ep5 into CsaB
ep5R	CCCTCACCATAATTGTTACCTCCATTACCACCGGAtgacgttccaaaatttaat	Paired w/ CsaB-F to insert ep5 into CsaB
ep6F	GGCTACGGTGGTAATGCTATCACAGGAGATAACttagtgattggtgcgactac	Paired w/ CsaB-R to insert ep6 into CsaB
ep6R	ATAGCATTACCACCGTAGCCATCATAtgacgttccaaaatttaatt	Paired w/ CsaB-F to insert ep6 into CsaB
ep7F	AGGTAAAGAAGATGATGGAACACAAAATGTAttagtgattggtgcgactac	Paired w/ CsaB-R to insert ep7 into CsaB
ep7R	GTTCCATCATCTTCTTTACCTGAAATTATATAtgacgttccaaaatttaatt	Paired w/ CsaB-F to insert ep7 into CsaB
ep8F	ATATCAGTGACCAGAATAAATTGATCTACGGTttagtgattggtgcgactac	Paired w/ CsaB-R to insert ep8 into CsaB
ep8R	TTTATTCTGGTCACTGATATAGCTTTTTGTtgacgttccaaaatttaatt	Paired w/ CsaB-F to insert ep8 into CsaB
ep9F	TGATACAGATGGCGACAGTCATGGAttagtgattggtgcgactac	Paired w/ CsaB-R to insert ep9 into CsaB
ep9R	GACTGTCGCCATCTGTATCATTCCAtgacgttccaaaatttaatt	Paired w/ CsaB-F to insert ep9 into CsaB
ep10F	GCAGATAATCTCAGCCATCATAATATAAATttagtgattggtgcgactac	Paired w/ CsaB-R to insert ep10 into CsaB
ep10R	TGATGGCTGAGATTATCTGCCAGAATAGTACTtgacgttccaaaatttaatt	Paired w/ CsaB-F to insert ep10 into CsaB
ep11F	GGAACTCTCATTTTGGCGGAAAAAAATACCTACttagtgattggtgcgactac	Paired w/ CsaB-R to insert ep11 into CsaB
ep11R	TCCGCCAAAATGAGAGTTCCCTCCCCTGATTTTGTtgacgttccaaaatttaatt	Paired w/ CsaB-F to insert ep11 into CsaB
CsaB-F	CTAGCTAGCGTAGAGAAAAATATCACTGTAA	Forward primer to amplify CsaB-VirG epitope fusion gene
CsaB-R	TCACGGCCGTTATTATGATGCTAAGGTCATTAA	Reverse primer to amplify CsaB-VirG epitope fusion gene
CotD/VirG_81-192_R	ATTCCATCTTCACCAGCTCCCAAATCTGATTCATATAAAAATCTCT	Paired w/ CotDF to insert VirG_81–192_ into CotD
VirG_81-192_F	TTGGGAGCTGGTGAAGATGGAATGGATGCGTGGTA	Amplification of VirG_81–192_
VirG_81-192_R	ATAACTATCGCCACCATGTCCACCTGAAATAATTT	Amplification of VirG_81–192_
CotD/VirG_81-192_F	GGTGGACATGGTGGCGATAGTTATGGTAAATATTCTGGTAGTAAC	Paired w/ CotDR to insert VirG_81–192_ into CotD
CotD/VirG_216-326_R	CTCCATTACCACCGGAAATAGTATCTGATTCATATAAAAATCTCTTA	Paired w/ CotDF to insert VirG_216–326_ into CotD
VirG_216-326_F	ACTATTTCCGGTGGTAATGGAGGTAACAATTATGGTGAGG	Amplification of VirG_216–326_
VirG_216-326_R	AGCATTACCTGCTACATTTTGTGTTCCATCATCTTCTTTA	Amplification of VirG_216–326_
CotD/VirG_216-326_F	GGAACACAAAATGTAGCAGGTAATGCTGGTAAATATTCTGGTAGTAA	Paired w/ CotDR to insert VirG_216–326_ into CotD
CotD/VirG_466-544_R	CTGATATAGCTTTTTGTATAGTCAATATCTGATTCATATAAAAATC	Paired w/ CotDF to insert VirG_466–544_ into CotD
VirG_466-544_ F	ATTGACTATACAAAAAGCTATATCAGTGACCAGAATAAAT	Amplification of VirG_466–544_
VirG_466-544_ R	AGAGTAGGTATTTTTTTCCGCCAAAATGAGAGTTCCCTCC	Amplification of VirG_466–544_
CotD/VirG_466-544_F	GGCGGAAAAAAATACCTACTCTGGTAAATATTCTGGTAGTAACGTGC	Paired w/ CotDR to insert VirG_466–544_ into CotD
CotDF	CGGGCTAGCGGTTCAATAAATAAAACAGAGTCG	Forward and reverse primers for CotD/VirG fusions
CotDR	TTACGGCCGTTATTACAGACTTGAACTACTAGGAGTAA	

^*a*^ For the primers in PCRs to insert VirG epitope sequences into CsaB, nucleotides in uppercase are of the VirG epitopes, and those in lowercase are of carrier CsaB. The nucleotides underlined are of a restriction enzyme site.

### *Shigella* VirG epitope fusion proteins were strongly immunogenic

**Fig 2:**
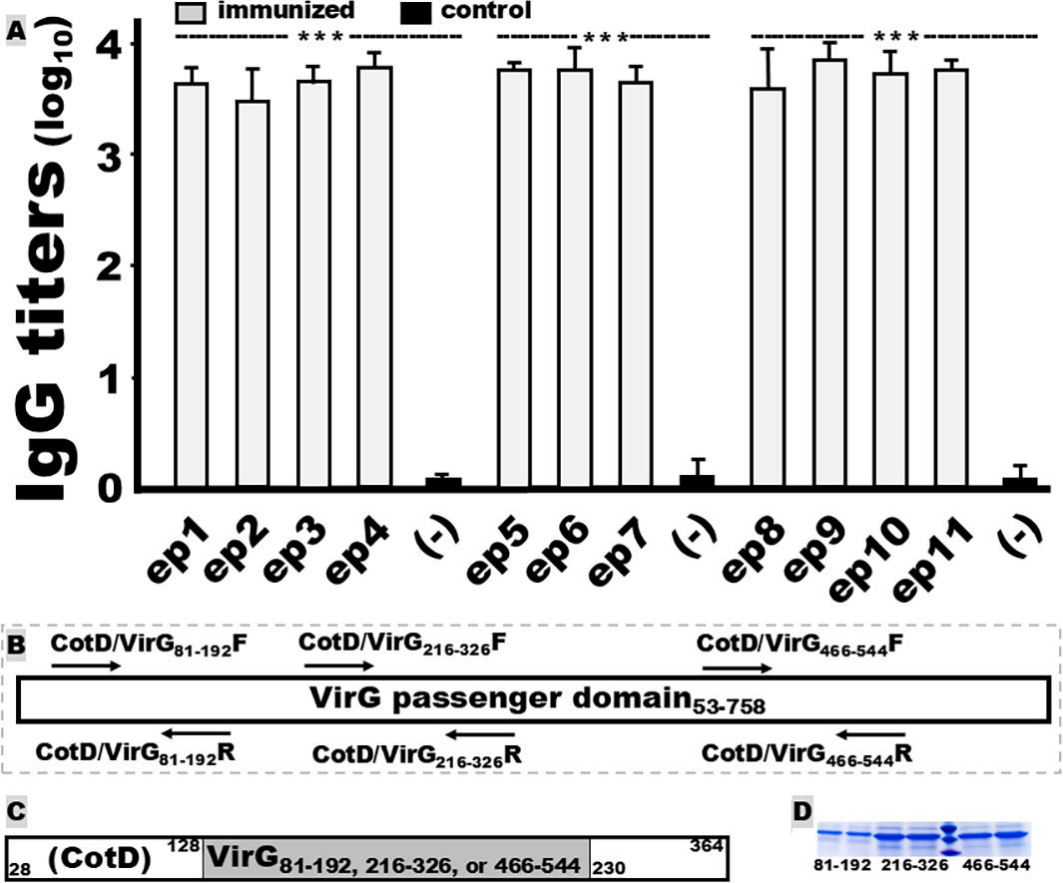
Mouse serum IgG titers (log_10_) after intramuscular immunization with VirG epitope fusion proteins. (**A**) IgG titers from mouse sera, with CotD-VirG_81–192_, CotD-VirG_216–326_, or CotD-VirG_466–544_ recombinant protein as the coating antigen in ELISAs to detect IgG responses from epitope fusion 1–4, 5–7, and 8–11, respectively. Boxes and bars indicate IgG titer means and standard deviations. *** indicates a *P* value of <0.001. (**B**) A schematic diagram shows three pairs of PCR primers used to amplify three VirG segments: VirG_81–192_, VirG_216–326_, and VirG_466–544_. (**C**) A schematic diagram shows the insertion of each VirG segment into carrier CotD for CotD-VirG fusion proteins CotD-VirG_81–192_, CotD-VirG_216–326_, and CotD-VirG_466–544_, which were used the ELISA coating antigens. CotD is the adhesive subunit of enterotoxigenic *E. coli* adhesin CS2. (**D**) SDS-PAGE detection of three CotD-VirG proteins (in duplicate) with Coomassie blue staining. Molecular mark bands from top to bottom: 50, 37, and 25 kDa.

Mice intramuscularly immunized with each VirG epitope fusion protein developed robust antigen-specific IgG responses (Fig. 2A). Because of the difficulty in expressing the entire VirG or the VirG passage domain gene, we amplified three segments from the VirG passage domain to cover epitopes 1–4, 5–7, and 8–11 separately (Fig. 2B), with specific PCR primers (Table 1). Each segment was inserted into the gene encoding CotD, a non-homologous adhesin subunit of *E. coli* adhesin CS1 (Fig. 2C), in PCRs with specific primers (Table 1). Three CotD-VirG fusion proteins, at ~37.5 kDa (Fig. 2D), were expressed and used as enzyme-linked immunosorbent assay (ELISA) coating antigens to titrate IgG specific for the epitope fusions, respectively.

IgG titers (log_10_) detected from the serum dilutions of each mouse immunized with the EP1, EP2, EP3, EP4, EP5, EP6, EP7, EP8, EP9, EP10, or EP11 fusion protein were 3.7 ± 0.2, 3.5 ± 0.3, 3.7 ± 0.2, 3.8 ± 0.1, 3.8 ± 0.1, 3.8 ± 0.2, 3.7 ± 0.2, 3.6 ± 0.3, 3.9 ± 0.2, 3.7 ± 0.2, and 3.8 ± 0.1 (log_10_), respectively. IgG titers in the mice intramuscularly immunized with CotD-VirG fragment 1, 2, or 3 were 4.3 ± 0.36, 4.5 ± 0.27, and 4.3 ± 0.55, respectively (log_10_).

No IgG responses were detected from the sera of the control mice or any sera collected before the prime. No IgA responses were detected from the control or the immunized mouse sera. No mouse serum samples reacted to the carrier protein (CotD) of the VirG ELISA coating antigens (CotD-VirG segments).

### *Shigella* VirG epitope-derived mouse serum antibodies inhibited *S*. *sonnei* and *S*. *flexneri* 2a invasion *in vitro*

**Fig 3:**
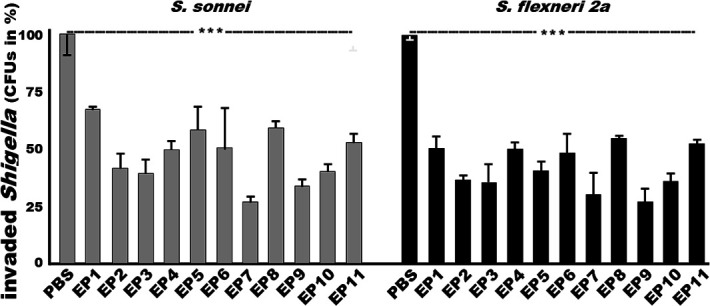
*S. sonnei* and *S. flexneri* 2a bacterial (CFUs, in %) invasion into HeLa cells after treatment with sera of the mice immunized with VirG epitope fusion proteins, based on antibody invasion inhibition assays. *Shigella* bacteria (2 × 10^7^ CFUs) after incubation with mouse sera (30 µL, heat-inactivated, pooled from each group intramuscularly immunized with an individual epitope fusion protein) were transferred to and incubated with HeLa cells (~2.5 × 10^5^), then treated with gentamycin to remove extracellular bacteria, lysed, diluted, and plated on agar plates for overnight culture. Invaded bacterial CFUs were converted to percentage based on the invaded bacteria treated with the control sera as 100%. Boxes and bars indicate CFU means and standard deviations; *** for *P* < 0.001.

After incubation with the sera from the mice immunized with the VirG EP1, EP2, EP3, EP4, EP5, EP6, EP7, EP8, EP9, EP10, or EP11 fusion protein, *S. sonnei* bacterial invasion to HeLa cells was reduced by 33.3 ± 0.7%, 58.7 ± 6.8%, 61.4 ± 7.4%, 49.2 ± 3.5%, 42 ± 10.2%, 48.5 ± 18.4%, 72.6 ± 2.7%, 39.3 ± 2.7%, 65.8 ± 3.4%, 59.8 ± 3.4%, and 47.6 ± 4.1%, respectively, significantly lower than the invasion by the same bacteria after incubation with the control mouse sera (100%) (Fig. 3, left). Antibodies derived from epitope #7, #9, #3, #2, or #10 were more effective against *S. sonnei* invasion *in vitro*.

Similarly, after incubation with the sera from the mice immunized with the EP1, EP2, EP3, EP4, EP5, EP6, EP7, EP8, EP9, EP10, or EP11 fusion protein, *S. flexneri* 2a bacterial invasion to HeLa cells was reduced by 50.1 ± 5.6%, 64.4 ± 2.2%, 65.5 ± 8.3%, 49.8 ± 3.1%, 60.2 ± 4.1%, 52.4 ± 12.0%, 69.9 ± 9.9%, 46.2 ± 1.5%, 73 ± 5.5%, 64.5 ± 3.0%, and 47.2 ± 1.8%, respectively (Fig. 3, right). Antibodies derived from epitope #9, #7, #3, #2, or #10 appeared more effective against *S. flexneri* 2a invasion *in vitro*.

### *Shigella* VirG epitope-derived mouse serum antibodies inhibited *S*. *flexneri* 2a bacterial adherence *in vitro*

To further assess serum antibodies from the epitope fusions for functional activity against *Shigella* bacterial adherence, we incubated bacteria with the mouse sera that were more effective against invasion with *S. flexneri* 2a, applied them to HeLa cells, and prepared two groups, with or without gentamicin treatment. After incubation, one group was treated with gentamicin to eliminate extracellular *Shigella* bacteria; thus, only the invaded bacteria were counted. The other group received no gentamicin treatment; therefore, both adhered and invaded *Shigella* bacteria recovered. By subtracting the invaded bacteria from the adhered and invaded bacteria, the adhered *Shigella* bacteria were calculated.

**Fig 4:**
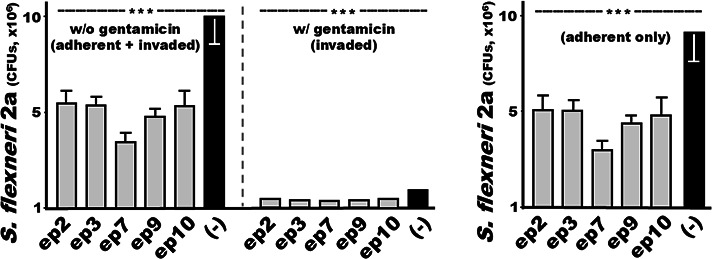
*S. flexneri* 2a bacterial (CFUs, in %) adherence and/or invasion to HeLa cells after treatment with sera of the mice immunized with fusion proteins with VirG epitope #2, #3, #7, #9, or #10. *S. flexneri* 2a bacteria (1.8 × 10^8^ CFUs) after incubation with mouse sera (30 µL, pooled from each group immunized with an individual fusion protein, heat-inactivated) were transferred to and incubated with HeLa cells (~2.5 × 10^5^). After incubation, one group was treated with gentamicin to calculate the number of invaded bacteria, and the other was not to measure the number of adherent and invaded bacteria. Subtraction of the invaded bacteria from the adhered and invaded bacteria resulted in the adherent bacteria (CFUs). Boxes and bars indicate CFU means and standard deviations; *** for *P* < 0.001.

After incubation with the sera from the mice immunized with the fusion of epitope #2, #3, #7, #9, or #10, the numbers of *S. flexneri* 2a adhered to HeLa cells were 5.0 ± 0.84, 5.0 ± 0.56, 3.0 ± 0.56, 4.4 ± 0.43, and 4.8 ± 0.91 (CFUs, ×10^6^), respectively. The adherent bacteria were reduced by 44%, 44.4%, 66.5%, 51.4%, and 47%, compared to the adherent bacteria after incubation with the control sera (9.0 ± 1.54; *P* < 0.001) (Fig. 4).

### *Shigella* VirG epitope #7 ranked the top for inducing functional antibodies against *Shigella* bacterial invasion and adherence

Fusion proteins with VirG epitopes #2, #3, #7, #9, or #10 were more effective in inducing functional antibodies against *S. sonnei* or *S. flexneri* 2a invasion. The fusion with epitope #7 was the most effective against *S. sonnei* invasion. For antibody invasion inhibition against *S. flexneri* 2a, fusions with epitope #9 or #7 were more effective. Additionally, the fusion with epitope #7 was the most effective against *S. flexneri* 2a adherence among the five epitope fusions. The epitope #7 became the top candidate to represent VirG for optimal *Shigella* MEFA construction and cross-protective vaccine development.

## DISCUSSION

Results from the current study indicated that all 11 B-cell epitopes of the VirG passenger domain induced robust IgG responses, but these epitope-specific antibodies varied in *in vitro* protection against bacterial adherence and invasion by *S. sonnei* and *S. flexneri* 2a. IgG antibodies to the VirG or its passenger domain were reported previously to be correlated to protection against *Shigella* infection clinically or preclinically (54, 56). This study identified the functional epitopes of the passenger domain that induce protective antibodies against *Shigella* bacterial *in vitro* adherence and invasion. The VirG functional epitopes identified from this study, together with the functional epitopes from IpaB and IpaD identified empirically in previous studies (38, 39), and Shiga toxins (unpublished data), enable us to construct an optimal epitope- and structure-based *Shigella* MEFA protein to induce broadly protective antibodies against the key *Shigella* virulence determinants and develop a cross-protective vaccine against shigellosis.

VirG is a multifunctional virulence factor that is critical in *Shigella* infection. At the early stage of infection, VirG acts as an adhesin to facilitate *Shigella* bacterial adherence to host receptor(s) and also as an invasin since VirG-dependent adhesion enhances *S. flexneri* bacterial invasion to host cells (43). After entering host cells, VirG facilitates the formation of actin tails and ABM for *Shigella* intercellular and intracellular spreading (57). The VirG passenger domain contains peptide domains associated with bacterial adherence, invasion, spread, and other roles (55). Among the eleven immunodominant B-cell epitopes investigated in this study, epitopes #2–#7 are from the GRR (117–332); GRR interacts with N-WASP to form actin tails for intercellular and intracellular spread (47, 58). Epitope #8 is located at the VirG insertion domain (VID; 422–483); VID might be another auto-chaperone to form VirG into different conformation states (55). Epitopes #10 and #11 are in the region (507–758) to interact with an important *Shigella* enzyme that plays a role in bacterial intercellular spread (59). Epitope #11 is also in the passenger-associated transport repeat (PATR; 526–557), which is involved in the outer membrane secretion of the VirG passenger domain (60).

Data from this study indicated that antibodies derived from the fusion with epitope #2, #3, #7, #9, or #10 appeared more effective than the other epitopes at inhibiting *S. sonnei* and *S. flexneri* 2a invasion and *S. flexneri* 2a adherence, suggesting potential roles played by these epitopes in *Shigella* adherence and invasion. It is not unexpected that VirG epitopes #2 and #3 effectively induced functional antibodies against *Shigella* invasion and adherence since a peptide of residues from 138 to 148, right between epitopes #2 and #3, was reported earlier to play a role in *Shigella* adherence and intracellular spreading (44). Disrupting the 138–148 peptide with either a deletion or an insertion (with five amino acid residues, though a deletion or an insertion could result in an alteration of structural conformation or antigenic topology) significantly reduced *Shigella* bacterial adherence. Specific antibodies recognizing either #2 or #3 epitope will likely dramatically affect the role played by the flanking peptide 138–148 on *Shigella* adherence and intracellular spreading. Moreover, the 130th residue cysteine (C_130_) of VirG, which resides inside epitope #2, was demonstrated to be important for *Shigella* bacterial adherence (61); thus, antibodies to epitope #2 were expected to inhibit *Shigella* adherence. Interestingly, this 138–148 peptide, or the C_130_, was reported as not important for *Shigella* invasion, indicated by the fact that this peptide did not react with functional anti-IcsA antibodies, and the mutation of C_130_ did not affect *Shigella* bacterial invasion (44, 61). Our results showed that sera from the mice immunized with epitope #2 or #3 fusion significantly reduced *Shigella* invasion and adherence, though the decrease in *Shigella* adherence from the antibodies derived from epitope #2 or #3 could be correlated with a reduction of bacterial invasion.

Mouse serum antibodies derived from the fusion with epitope #9 or #10 were also effective against *S. sonnei* or *S. flexneri* 2a invasion and adherence. However, neither epitope #9 nor #10 is aligned with any VirG domains with known functions or biogenesis. In contrast, epitope #8 is in the VID, and epitope #11 is a part of the PATR and also the polar targeting region (PT), which are expected to be important for VirG biogenesis. However, our data showed that epitopes #9 and #10 are effective in inducing functional antibodies against *Shigella* invasion and adherence, whereas epitopes #8 and #11 are less critical for the induction of functional antibodies. That suggests that some VirG peptides or epitopes may play antigenic and biogenic roles separately.

Epitope #7 (_309_YIISGKEDDGTQNV_325_), on the other hand, is important both biogenetically and antigenically. This epitope is within the VirG GRRs and the ATG5/IcsB binding region. The GRR region is required for the formation of actin tails to propel *Shigella* bacterial movement intercellularly and intracellularly (58); VirG ATG5/IcsB binding region binds both ATG5 (a human autophagy protein) and IcsB and plays a critical role in escaping *Shigella* bacteria from degradation by autophagy (62). Thus, epitope #7 can be important for VirG biogenesis and *Shigella* infection. Antigenically, antibodies induced by the fusion with the epitope #7 are the most effective against *S. sonnei* invasion, the second best against *S. flexneri* 2a invasion, and the most effective against *S. flexneri* 2a adherence *in vitro*. That makes epitope #7 the top candidate to represent VirG as a *Shigella* vaccine antigen component. Since VirG is an adhesin and an invasin, antibodies derived from this VirG epitope are expected to be effective against *Shigella* invasion and adherence. However, whether functional antibodies from epitope #7 will negatively affect comet-like actin tail formation and ABM, as well as escape from autophagy degradation *in vivo* is currently unknown. Additionally, whether antibodies derived from epitope #7 remain functional *in vivo* or are also effective against invasion and adherence of other *Shigella* species and serotypes is to be confirmed in future studies.

The limitation of the current study included that we only examined antibody functions *in vitro* against *S. sonnei* and *S. flexneri* 2a. Future studies will be needed to confirm antibody activities against invasion and adherence for *S. flexneri* 3a, 6, and 1b, as well as *S. boydii* and *S. dysentariae*, and, more importantly, antibody *in vivo* protection against *Shigella* infection. Additionally, we only cloned the VirG segments covering the target epitopes because of the difficulty in cloning and expressing the entire VirG gene or the passenger domain. Though using the shortened segment as ELISA coating antigens is unlikely to alter the outcomes of epitope-specific antibody titration, a full-length passenger domain protein as the coating antigen for antibody titration or using sera from mice immunized with the entire alpha domain as a positive control (perhaps from other research groups) in antibody function assays will be more informative. Nevertheless, results from this study provide us with the needed information to construct an optimal polyvalent antigen for a cross-protective vaccine against shigellosis.

## MATERIALS AND METHODS

### Bacterial strains and plasmids

Bacterial strains and plasmids used in this study are listed in Table 2. *S. sonnei* and *S. flexneri* 2a used in the antibody function assays were provided by Drs. D. A. Sack at Johns Hopkins University and E. M. Barry at the University of Maryland, Baltimore. Vector pET28a (Novagen, Madison, WI, USA) was used to clone the VirG epitope fusion genes and three VirG (CotD-VirG) passenger domain segments. *E. coli* DH5α (Promega, Madison, WI, USA) and BL21 (DE3) (Agilent, Santa Clara, CA, USA) were used to host or express each epitope fusion or the VirG segments.

**TABLE 2:** Bacterial strains and plasmids used in the study^*a*^

Strain	Characteristics	Source/reference
*E. coli* DH5α	*fhuA2* Δ(*argF-lacZ*) U169 *phoA glnV44* φ80 Δ(*lacZ*)M15 *gyrA96 recA1 relA1 endA1 thi-1 hsdR17*	Promega
*E. coli* BL21(DE3)	*huA2* Δ(*argF-lacZ*) U169 *phoA glnV44* φ*80* Δ(*lacZ*)M15 *gyrA96 recA1 relA1 endA1 thi-1 hsdR17*	Agilent
9766	CS4 major subunit (CsaB) in pET28α/*E. coli* BL21	(34)
9843	VirG-epitope-1-CsaB in pET28α/*E. coli* BL21	This study
9833	VirG-epitope-2-CsaB in pET28α/*E. coli* BL21	This study
9834	VirG-epitope-3-CsaB in pET28α/*E. coli* BL21	This study
9835	VirG-epitope-4-CsaB in pET28α/*E. coli* BL21	This study
9836	VirG-epitope-5-CsaB in pET28α/*E. coli* BL21	This study
9837	VirG-epitope-6-CsaB in pET28α/*E. coli* BL21	This study
9838	VirG-epitope-7-CsaB in pET28α/*E. coli* BL21	This study
9839	VirG-epitope-8-CsaB in pET28α/*E. coli* BL21	This study
9840	VirG-epitope-9-CsaB in pET28α/*E. coli* BL21	This study
9841	VirG-epitope-10-CsaB in pET28α/*E. coli* BL21	This study
9842	VirG-epitope-11-CsaB in pET28α/*E. coli* BL21	This study
9992	VirG_(81–192)_ in pET28α/*E. coli* BL21	This study
9993	VirG_(216–326)_ in pET28α/*E. coli* BL21	This study
9994	VirG_(466–544)_ in pET28α/*E. coli* BL21	This study
9904	*Shigella sonnei* 53G	Johns Hopkins University
9905	*Shigella flexneri* 2457T	University of Maryland
Plasmids		
pET28α	Protein expression vector	Novagen

^*a*^ *E. coli* DH5α was used to host the plasmid carrying each VirG epitope fusion gene, and BL21 (DE3) Codon Plus strain was used to express epitope fusion and VirG segment recombinant proteins. *Shigella* strains *S. sonnei* 53g and *S. flexneri 2a* 2457T were used for antibody function assays. Plasmid 9765, ETEC adhesin CS4 major subunit CsaB gene cloned in pET28α vector, was used as the carrier to construct VirG epitope fusion genes. ETEC adhesin CS2 subunit CotD was used as the carrier for VirG recombinant proteins used as ELISA coating antigens for antibody titration.

### VirG passenger domain epitope *in silico* prediction and selection

Program of sequence-based B-cell epitope prediction using conformational epitopes, BepiPred 2.0 (63), was used to *in silico* identify epitopes from the VirG functional passenger domain (VirG_53–758_), with amino acid sequence (accession NP_858315.1) as the template. Predicted epitopes were examined for homology across *S. sonnei* (53G), *S. flexneri* 2a (2457T), 3a (J17A), 6 (CCHO60), *S. boydii* (NCTC 12985), and *S. dysenteriae SD197*. A panel of top-ranked epitopes was selected based on antigenicity scores. The selected epitopes were visually assessed for surface presentation and locations at the VirG protein 3-D model created with Protein Homology/AnalogY Recognition Engine version 2.0 (Phyre 2) (64). The presentation of each VirG epitope on the epitope fusion protein was also assessed using Phyre 2.

### VirG passenger domain epitope fusion construction, expression, and characterization

Enterotoxigenic *E. coli* (ETEC) adhesin CS4 subunit CsaB (17 kDa; anti-CsaB sera showed no reactivity with recombinant VirG segment proteins) was used as the carrier to present each VirG epitope for epitope fusions. Nucleotides of each VirG epitope were inserted into the *CsaB* gene in overlapping PCR with primers listed in Table 1. Each epitope fusion gene was cloned into the vector pET28a and hosted by *E. coli* DH5α strain. Because of the difficulty in expressing the entire VirG passenger domain protein, we PCR amplified three segments of the passenger domain, inserted them into ETEC CS2 adhesin tip subunit CotD, and cloned them into pET28a. Since these three VirG segments were used as ELISA coating antigens, a different carrier (CotD, not the CsaB that was used for epitope fusions) was used to detect epitope-specific immune responses.

The epitope fusions and the VirG passenger domain segments were expressed in *E. coli* BL21 (DE3) strain. As described previously (33, 38, 39, 65), a single colony of each recombinant strain was inoculated in Luria-Bertani (LB) broth supplemented with kanamycin (30 µg/mL) and cultured at 37°C overnight. The overnight-grown inoculum was sub-cultured in 2× YT broth supplemented with kanamycin (30 µg/mL) at 37°C in a shaker incubator (200 rpm) till OD_600_ reached 0.5 –0.7, then induced with 1 mM isopropyl-β-d-1-thiogalactopyranoside (IPTG; Sigma, St. Louis, MO, USA) and cultured for 4 h. Bacteria were harvested with centrifugation (13,000 × *g*) for 15 min at 4°C and stored at a −80°C freezer.

Bacterial pellets were thawed on ice and suspended in 10 mL bacterial protein extraction reagent (B-PER, in phosphate buffer, pH 7.5; Thermo Fisher Scientific, Rochester, NY, USA). The resuspension was treated with lysozyme (200 µg/mL) and sonicated at 130 W, 20 kHz for 15 min, with an Ultrasonic processor GEX130 (Cole Parmer, Vernon Hills, IL, USA). Bacterial lysates were used to extract inclusion body proteins by following the manufacturer’s protocol (B-PER). Extracted proteins were then solubilized in solubilization buffer, 50 mM CAPS [3-(cyclohexylamine) propane sulfonic acid, pH 11.0; Sigma], 0.3% N-lauroylsarcosine, and 1 mM dithiothreitol (DTT). The solubilized protein was transferred to a 6–8 kDa Spectrum membrane tubing (Fisher Scientific) and refolded in a refolding and dialysis buffer (1 M Tris-HCl, pH 8.5) at 4°C with two buffer changes (8 h each). Refolded proteins were collected and examined on sodium dodecyl sulfate-polyacrylamide gel electrophoresis (SDS-PAGE) with Coomassie blue staining.

### Mouse intramuscular immunization with VirG epitope fusion proteins

Eight-week-old BALB/c female mice (Charles River Laboratories, Wilmington, MA, USA), five per group, were administered intramuscularly with 40 µg of each epitope fusion protein (in 40 µL PBS) or PBS (a negative control), with 0.2 µg double mutant heat-labile toxin (dmLT, LT_R192G/L211A_; provided by Dr. Jacob Bitoun at Tulane University) as the adjuvant. Two boosters, 2 weeks apart, were administered after the primary. Mice were euthanized 2 weeks after the second booster. Blood samples were collected via cardiac puncture, and sera were prepared and stored at −80°C until use.

The three CotD-VirG segments were also used in mouse intramuscular immunization.

### Mouse serum antibody titration

Mouse sera were examined for antibody responses to VirG in ELISA as described previously (33, 38, 39). Briefly, 96-well Immulon 2-HB plates (Thermo Fisher Scientific) were coated with a CotD-VirG segment recombinant protein (CotD-VirG_81–192_, CotD-VirG_216–326,_ or CotD-VirG_466–544_ fusion protein), 100 ng per well, in 100 µL bicarbonate/carbonate antigen coating buffer (50 mM), at 37°C for 1 h, and then 4°C overnight. Coated wells were washed with phosphate-buffered saline with 0.05% Tween-20 (PBST), and blocked uncoated spots with 10% skim milk (in PBST). Serum from each mouse was twofold diluted (1:400 to 1:25,600); serum dilutions, in triplicate, were added to wells and incubated for 1 h at 37°C. Horseradish peroxidase-conjugated goat anti-mouse IgG or IgA secondary antibody (Sigma) and 3,3,5,5-tetramethylbenzidine substrate (KPL, Gaithersburg, MD, USA) were used to measure optical density (OD) with a SPECTROstar^Nano^ spectrometer (BMG LABTECH, Germany) at a wavelength of 650 nm. After subtraction of background readings, the highest dilution that gave an OD_650_ above 0.3 was used (OD × dilution) to calculate antibody titers. Antibody titers were presented at the log_10_ scale.

### Mouse serum antibody invasion inhibition assay

Sera from the mice immunized with each epitope fusion were examined for activity against *Shigella* bacterial invasion as described previously (33, 38, 39). Briefly, *S. sonnei* or *S. flexneri* 2a Congo red strain bacteria (~ 2 × 10^7^ CFUs in 30 µL PBS) were mixed with 30 µL heat-inactivated (56°C for 30 min) mouse sera, which were pooled from each group, and incubated at room temperature for 25 min, and then transferred to >95% confluent monolayered HeLa cells (ATCC, CCL-2) on a 24-well culture plate. Plates were centrifuged at 1,000 × *g* for 10  min and incubated for 2 h at 37°C. The cells were rinsed with 1× PBS to remove non-adherent *Shigella* bacteria and treated with gentamycin (300 µg/mL, Sigma) in Dulbecco’s modified Eagle’s medium (Thermo Fisher) for 2 h to eliminate extracellular bacteria. The cells were washed and treated with a 0.5% Triton X-100 solution to release the invaded *Shigella* bacteria. Cell lysates were collected, serially diluted in PBS, plated on LB agar plates, and cultured at 37°C overnight. Bacterial CFUs were counted and converted to percentages, with the CFUs treated with the control sera set as 100%.

### Mouse serum antibody adherence inhibition assay

Antibody *in vitro* adherence inhibition assay protocol was adopted from the one against ETEC or *Vibrio cholerae* (65, 66). Two sets of HeLa cells were prepared to examine mouse serum antibody activity against *Shigella* bacterial adherence. *S. flexneri* 2a (1.8 × 10^8^ CFUs), after incubation with mouse serum samples (heat-inactivated) at room temperature for 1 h, were transferred to HeLa cells, using the same procedure as for antibody invasion inhibition assays described above. Afterward, one set incubated with gentamicin (to eliminate extracellular bacteria) was used to quantify the invaded bacteria, and the other set without gentamicin treatment was used to measure the adhered and invaded *Shigella* bacteria. After washing with PBS, HeLa cells were dislodged, collected, serially diluted, plated on agar plates, cultured overnight at 37°C, and the CFUs were counted. Subtraction of the invaded (intracellular) bacteria (CFUs) from the adhered and invaded bacteria allowed us to calculate the adherent (extracellular) *Shigella* bacteria.

### Statistical analysis

GraphPad Prism Version 7 (GraphPad Software, San Diego, CA, USA) was used for statistical analyses. The difference in antibody titers, antibody invasion inhibition, and antibody adherence inhibition assays between treatment groups was examined using Bonferroni’s multiple comparisons test and two-way analysis of variance. A *P* value < 0.05 indicated a statistically significant difference.

## References

[1] Kotloff KL, Nataro JP, Blackwelder WC, Nasrin D, Farag TH, Panchalingam S, Wu Y, Sow SO, Sur D, Breiman RF, et al.. 2013. Burden and aetiology of diarrhoeal disease in infants and young children in developing countries (the Global Enteric Multicenter Study, GEMS): a prospective, case-control study. Lancet 382:209–222. doi:10.1016/S0140-6736(13)60844-2 23680352

[2] Levine MM, Kotloff KL, Barry EM, Pasetti MF, Sztein MB. 2007. Clinical trials of Shigella vaccines: two steps forward and one step back on a long, hard road. Nat Rev Microbiol 5:540–553. doi:10.1038/nrmicro1662 17558427 PMC3771495

[3] Akhondi H, Goldin J, Simonsen KA. 2025. Bacterial diarrhea. StatPearls, Treasure Island (FL) ineligible companies. 31869107

[4] Mani S, Wierzba T, Walker RI. 2016. Status of vaccine research and development for Shigella. Vaccine (Auckl) 34:2887–2894. doi:10.1016/j.vaccine.2016.02.075 26979135

[5] Hale TL. 1991. Genetic basis of virulence in Shigella species. Microbiol Rev 55:206–224. doi:10.1128/mr.55.2.206-224.1991 1886518 PMC372811

[6] McKenzie R, Venkatesan MM, Wolf MK, Islam D, Grahek S, Jones AM, Bloom A, Taylor DN, Hale TL, Bourgeois AL. 2008. Safety and immunogenicity of WRSd1, a live attenuated Shigella dysenteriae type 1 vaccine candidate. Vaccine (Auckl) 26:3291–3296. doi:10.1016/j.vaccine.2008.03.079 18468742

[7] Launay O, Sadorge C, Jolly N, Poirier B, Béchet S, van der Vliet D, Seffer V, Fenner N, Dowling K, Giemza R, Johnson J, Ndiaye A, Vray M, Sansonetti P, Morand P, Poyart C, Lewis D, Gougeon M-L. 2009. Safety and immunogenicity of SC599, an oral live attenuated Shigella dysenteriae type-1 vaccine in healthy volunteers: results of a Phase 2, randomized, double-blind placebo-controlled trial. Vaccine (Auckl) 27:1184–1191. doi:10.1016/j.vaccine.2008.12.021 19135496

[8] Kotloff KL, Simon JK, Pasetti MF, Sztein MB, Wooden SL, Livio S, Nataro JP, Blackwelder WC, Barry EM, Picking W, Levine MM. 2007. Safety and immunogenicity of CVD 1208S, a live, oral ΔguaBA Δsen Δset Shigella flexneri 2a vaccine grown on animal-free media. Hum Vaccin 3:268–275. doi:10.4161/hv.4746 17938573

[9] Rahman KM, Arifeen SE, Zaman K, Rahman M, Raqib R, Yunus M, Begum N, Islam MS, Sohel BM, Rahman M, Venkatesan M, Hale TL, Isenbarger DW, Sansonetti PJ, Black RE, Baqui AH. 2011. Safety, dose, immunogenicity, and transmissibility of an oral live attenuated Shigella flexneri 2a vaccine candidate (SC602) among healthy adults and school children in Matlab, Bangladesh. Vaccine (Auckl) 29:1347–1354. doi:10.1016/j.vaccine.2010.10.035 21040694

[10] Chakraborty S, Harro C, DeNearing B, Bream J, Bauers N, Dally L, Flores J, Van de Verg L, Sack DA, Walker R. 2016. Evaluation of the safety, tolerability, and immunogenicity of an oral, inactivated whole-cell Shigella flexneri 2a vaccine in healthy adult subjects. Clin Vaccine Immunol 23:315–325. doi:10.1128/CVI.00608-15 26865592 PMC4820506

[11] Girardi P, Harutyunyan S, Neuhauser I, Glaninger K, Korda O, Nagy G, Nagy E, Szijártó V, Pall D, Szarka K, Kardos G, Henics T, Malinoski FJ. 2022. Evaluation of the safety, tolerability and immunogenicity of ShigETEC, an oral live attenuated Shigella-ETEC vaccine in placebo-controlled randomized phase 1 trial. Vaccines (Basel) 10:340. doi:10.3390/vaccines10020340 35214798 PMC8879453

[12] Orr N, Katz DE, Atsmon J, Radu P, Yavzori M, Halperin T, Sela T, Kayouf R, Klein Z, Ambar R, Cohen D, Wolf MK, Venkatesan MM, Hale TL. 2005. Community-based safety, immunogenicity, and transmissibility study of the Shigella sonnei WRSS1 vaccine in Israeli volunteers. Infect Immun 73:8027–8032. doi:10.1128/IAI.73.12.8027-8032.2005 16299296 PMC1307051

[13] McKenzie R, Walker RI, Nabors GS, Van De Verg LL, Carpenter C, Gomes G, Forbes E, Tian JH, Yang HH, Pace JL, Jackson WJ, Bourgeois AL. 2006. Safety and immunogenicity of an oral, inactivated, whole-cell vaccine for Shigella sonnei: preclinical studies and a Phase I trial. Vaccine (Auckl) 24:3735–3745. doi:10.1016/j.vaccine.2005.07.014 16095766

[14] Barnoy S, Jeong KI, Helm RF, Suvarnapunya AE, Ranallo RT, Tzipori S, Venkatesan MM. 2010. Characterization of WRSs2 and WRSs3, new second-generation virG(icsA)-based Shigella sonnei vaccine candidates with the potential for reduced reactogenicity. Vaccine (Auckl) 28:1642–1654. doi:10.1016/j.vaccine.2009.11.001 PMC2999844 19932216

[15] Pitisuttithum P, Islam D, Chamnanchanunt S, Ruamsap N, Khantapura P, Kaewkungwal J, Kittitrakul C, Luvira V, Dhitavat J, Venkatesan MM, Mason CJ, Bodhidatta L. 2016. Clinical trial of an oral live Shigella sonnei vaccine candidate, WRSS1, in Thai adults. Clin Vaccine Immunol 23:564–575. doi:10.1128/CVI.00665-15 27146000 PMC4933782

[16] Frenck RW, Baqar S, Alexander W, Dickey M, McNeal M, El-Khorazaty J, Baughman H, Hoeper A, Barnoy S, Suvarnapunya AE, Kaminski RW, Venkatesan MM. 2018. A Phase I trial to evaluate the safety and immunogenicity of WRSs2 and WRSs3; two live oral candidate vaccines against Shigella sonnei. Vaccine (Auckl) 36:4880–4889. doi:10.1016/j.vaccine.2018.06.063 PMC10559265 30037478

[17] Chu CY, Liu BK, Watson D, Szu SS, Bryla D, Shiloach J, Schneerson R, Robbins JB. 1991. Preparation, characterization, and immunogenicity of conjugates composed of the O-specific polysaccharide of Shigella dysenteriae type 1 (Shiga’s bacillus) bound to tetanus toxoid. Infect Immun 59:4450–4458. doi:10.1128/iai.59.12.4450-4458.1991 1937803 PMC259062

[18] Taylor DN, Trofa AC, Sadoff J, Chu C, Bryla D, Shiloach J, Cohen D, Ashkenazi S, Lerman Y, Egan W. 1993. Synthesis, characterization, and clinical evaluation of conjugate vaccines composed of the O-specific polysaccharides of Shigella dysenteriae type 1, Shigella flexneri type 2a, and Shigella sonnei (Plesiomonas shigelloides) bound to bacterial toxoids. Infect Immun 61:3678–3687. doi:10.1128/iai.61.9.3678-3687.1993 8359890 PMC281064

[19] Pozsgay V, Chu C, Pannell L, Wolfe J, Robbins JB, Schneerson R. 1999. Protein conjugates of synthetic saccharides elicit higher levels of serum IgG lipopolysaccharide antibodies in mice than do those of the O-specific polysaccharide from Shigella dysenteriae type 1. Proc Natl Acad Sci USA 96:5194–5197. doi:10.1073/pnas.96.9.5194 10220442 PMC21840

[20] Bélot F, Guerreiro C, Baleux F, Mulard LA. 2005. Synthesis of two linear PADRE conjugates bearing a deca- or pentadecasaccharide B epitope as potential synthetic vaccines against Shigella flexneri serotype 2a infection. Chemistry 11:1625–1635. doi:10.1002/chem.200400903 15669066

[21] Phalipon A, Tanguy M, Grandjean C, Guerreiro C, Bélot F, Cohen D, Sansonetti PJ, Mulard LA. 2009. A synthetic carbohydrate-protein conjugate vaccine candidate against Shigella flexneri 2a infection. J Immunol 182:2241–2247. doi:10.4049/jimmunol.0803141 19201878

[22] Kämpf MM, Braun M, Sirena D, Ihssen J, Thöny-Meyer L, Ren Q. 2015. In vivo production of a novel glycoconjugate vaccine against Shigella flexneri 2a in recombinant Escherichia coli: identification of stimulating factors for in vivo glycosylation. Microb Cell Fact 14:12. doi:10.1186/s12934-015-0195-7 25612741 PMC4308876

[23] Riddle MS, Kaminski RW, Di Paolo C, Porter CK, Gutierrez RL, Clarkson KA, Weerts HE, Duplessis C, Castellano A, Alaimo C, Paolino K, Gormley R, Gambillara Fonck V. 2016. Safety and immunogenicity of a candidate bioconjugate vaccine against Shigella flexneri 2a administered to healthy adults: a single-blind, randomized phase I study. Clin Vaccine Immunol 23:908–917. doi:10.1128/CVI.00224-16 27581434 PMC5139601

[24] Camacho AI, Irache JM, de Souza J, Sánchez-Gómez S, Gamazo C. 2013. Nanoparticle-based vaccine for mucosal protection against Shigella flexneri in mice. Vaccine (Auckl) 31:3288–3294. doi:10.1016/j.vaccine.2013.05.020 23727423

[25] Rossi O, Pesce I, Giannelli C, Aprea S, Caboni M, Citiulo F, Valentini S, Ferlenghi I, MacLennan CA, D’Oro U, Saul A, Gerke C. 2014. Modulation of endotoxicity of Shigella generalized modules for membrane antigens (GMMA) by genetic lipid A modifications: relative activation of TLR4 and TLR2 pathways in different mutants. J Biol Chem 289:24922–24935. doi:10.1074/jbc.M114.566570 25023285 PMC4155660

[26] Launay O, Ndiaye AGW, Conti V, Loulergue P, Sciré AS, Landre AM, Ferruzzi P, Nedjaai N, Schütte LD, Auerbach J, Marchetti E, Saul A, Martin LB, Podda A. 2019. Booster vaccination with GVGH Shigella sonnei 1790GAHB GMMA vaccine compared to single vaccination in unvaccinated healthy European adults: results from a phase 1 clinical trial. Front Immunol 10:335. doi:10.3389/fimmu.2019.00335 30906291 PMC6418009

[27] Turbyfill KR, Hartman AB, Oaks EV. 2000. Isolation and characterization of a Shigella flexneri invasin complex subunit vaccine. Infect Immun 68:6624–6632. doi:10.1128/IAI.68.12.6624-6632.2000 11083774 PMC97759

[28] Oaks EV, Turbyfill KR. 2006. Development and evaluation of a Shigella flexneri 2a and S. sonnei bivalent invasin complex (Invaplex) vaccine. Vaccine (Auckl) 24:2290–2301. doi:10.1016/j.vaccine.2005.11.040 16364513

[29] Tribble D, Kaminski R, Cantrell J, Nelson M, Porter C, Baqar S, Williams C, Arora R, Saunders J, Ananthakrishnan M, Sanders J, Zaucha G, Turbyfill R, Oaks E. 2010. Safety and immunogenicity of a Shigella flexneri 2a Invaplex 50 intranasal vaccine in adult volunteers. Vaccine (Auckl) 28:6076–6085. doi:10.1016/j.vaccine.2010.06.086 20619378

[30] Martinez-Becerra FJ, Kissmann JM, Diaz-McNair J, Choudhari SP, Quick AM, Mellado-Sanchez G, Clements JD, Pasetti MF, Picking WL. 2012. Broadly protective Shigella vaccine based on type III secretion apparatus proteins. Infect Immun 80:1222–1231. doi:10.1128/IAI.06174-11 22202122 PMC3294653

[31] Martinez-Becerra FJ, Chen X, Dickenson NE, Choudhari SP, Harrison K, Clements JD, Picking WD, Van De Verg LL, Walker RI, Picking WL. 2013. Characterization of a novel fusion protein from IpaB and IpaD of Shigella spp. and its potential as a pan-Shigella vaccine. Infect Immun 81:4470–4477. doi:10.1128/IAI.00859-13 24060976 PMC3837967

[32] Desalegn G, Tamilselvi CS, Lemme-Dumit JM, Heine SJ, Dunn D, Ndungo E, Kapoor N, Oaks EV, Fairman J, Pasetti MF. 2024. Shigella virulence protein VirG is a broadly protective antigen and vaccine candidate. NPJ Vaccines 9:2. doi:10.1038/s41541-023-00797-6 38167387 PMC10761965

[33] Li S, Anvari S, Ptacek G, Upadhyay I, Kaminski RW, Sack DA, Zhang W. 2023. A broadly immunogenic polyvalent Shigella multiepitope fusion antigen protein protects against Shigella sonnei and Shigella flexneri lethal pulmonary challenges in mice. Infect Immun 91:e0031623. doi:10.1128/iai.00316-23 37795982 PMC10652900

[34] Ruan X, Knudsen DE, Wollenberg KM, Sack DA, Zhang W. 2014. Multiepitope fusion antigen induces broadly protective antibodies that prevent adherence of Escherichia coli strains expressing colonization factor antigen I (CFA/I), CFA/II, and CFA/IV. Clin Vaccine Immunol 21:243–249. doi:10.1128/CVI.00652-13 24351757 PMC3910947

[35] Duan Q, Lee KH, Nandre RM, Garcia C, Chen J, Zhang W. 2017. MEFA (multiepitope fusion antigen)-novel technology for structural vaccinology, proof from computational and empirical immunogenicity characterization of an enterotoxigenic Escherichia coli (ETEC) adhesin MEFA. J Vaccines Vaccin 8:367. doi:10.4172/2157-7560.1000367 28944092 PMC5606245

[36] Li S, Lee KH, Zhang W. 2022. Multiepitope fusion antigen: MEFA, an epitope- and structure-based vaccinology platform for multivalent vaccine development, p 151–169. In Bidmos F, Bossé J, Langford P (ed), Methods in molecular biology. Vol. 2414. Springer Nature. doi:10.1007/978-1-0716-1900-1_10. PMC10294517 34784037

[37] Lu T, Moxley RA, Zhang W. 2019. Mapping the neutralizing epitopes of enterotoxigenic Escherichia coli (ETEC) K88 (F4) fimbrial adhesin and major subunit FaeG. Appl Environ Microbiol 85. doi:10.1128/AEM.00329-19 PMC6532040 30926730

[38] Li S, Han X, Upadhyay I, Zhang W. 2022. Characterization of functional B-cell epitopes at the amino terminus of Shigella invasion plasmid antigen B (IpaB). Appl Environ Microbiol 88:e0038422. doi:10.1128/aem.00384-22 35856689 PMC9361828

[39] Li S, Zhang W. 2024. Mapping the functional B-cell epitopes of Shigella invasion plasmid antigen D (IpaD). Appl Environ Microbiol 90:e0098824. doi:10.1128/aem.00988-24 39082807 PMC11337796

[40] Fukuda I, Suzuki T, Munakata H, Hayashi N, Katayama E, Yoshikawa M, Sasakawa C. 1995. Cleavage of Shigella surface protein VirG occurs at a specific site, but the secretion is not essential for intracellular spreading. J Bacteriol 177:1719–1726. doi:10.1128/jb.177.7.1719-1726.1995 7896693 PMC176798

[41] Suzuki T, Lett MC, Sasakawa C. 1995. Extracellular transport of VirG protein in Shigella. J Biol Chem 270:30874–30880. doi:10.1074/jbc.270.52.30874 8537341

[42] Brandon LD, Goldberg MB. 2001. Periplasmic transit and disulfide bond formation of the autotransported Shigella protein IcsA. J Bacteriol 183:951–958. doi:10.1128/JB.183.3.951-958.2001 11208794 PMC94963

[43] Brotcke Zumsteg A, Goosmann C, Brinkmann V, Morona R, Zychlinsky A. 2014. IcsA is a Shigella flexneri adhesin regulated by the type III secretion system and required for pathogenesis. Cell Host Microbe 15:435–445. doi:10.1016/j.chom.2014.03.001 24721572

[44] Qin J, Doyle MT, Tran ENH, Morona R. 2020. The virulence domain of Shigella IcsA contains a subregion with specific host cell adhesion function. PLoS One 15:e0227425. doi:10.1371/journal.pone.0227425 31910229 PMC6946128

[45] Bernardini ML, Mounier J, d’Hauteville H, Coquis-Rondon M, Sansonetti PJ. 1989. Identification of icsA, a plasmid locus of Shigella flexneri that governs bacterial intra- and intercellular spread through interaction with F-actin. Proc Natl Acad Sci USA 86:3867–3871. doi:10.1073/pnas.86.10.3867 2542950 PMC287242

[46] Lett MC, Sasakawa C, Okada N, Sakai T, Makino S, Yamada M, Komatsu K, Yoshikawa M. 1989. virG, a plasmid-coded virulence gene of Shigella flexneri: identification of the virG protein and determination of the complete coding sequence. J Bacteriol 171:353–359. doi:10.1128/jb.171.1.353-359.1989 2644195 PMC209595

[47] Goldberg MB, Bârzu O, Parsot C, Sansonetti PJ. 1993. Unipolar localization and ATPase activity of IcsA, a Shigella flexneri protein involved in intracellular movement. J Bacteriol 175:2189–2196. doi:10.1128/jb.175.8.2189-2196.1993 8468279 PMC204503

[48] Kocks C, Marchand JB, Gouin E, d’Hauteville H, Sansonetti PJ, Carlier MF, Cossart P. 1995. The unrelated surface proteins ActA of Listeria monocytogenes and IcsA of Shigella flexneri are sufficient to confer actin-based motility on Listeria innocua and Escherichia coli respectively. Mol Microbiol 18:413–423. doi:10.1111/j.1365-2958.1995.mmi_18030413.x 8748026

[49] Egile C, Loisel TP, Laurent V, Li R, Pantaloni D, Sansonetti PJ, Carlier MF. 1999. Activation of the CDC42 effector N-WASP by the Shigella flexneri IcsA protein promotes actin nucleation by Arp2/3 complex and bacterial actin-based motility. J Cell Biol 146:1319–1332. doi:10.1083/jcb.146.6.1319 10491394 PMC2156126

[50] Suzuki T, Sasakawa C. 2001. Molecular basis of the intracellular spreading of Shigella. Infect Immun 69:5959–5966. doi:10.1128/IAI.69.10.5959-5966.2001 11553531 PMC98722

[51] May KL, Morona R. 2008. Mutagenesis of the Shigella flexneri autotransporter IcsA reveals novel functional regions involved in IcsA biogenesis and recruitment of host neural Wiscott-Aldrich syndrome protein. J Bacteriol 190:4666–4676. doi:10.1128/JB.00093-08 18456802 PMC2446779

[52] Makino S, Sasakawa C, Kamata K, Kurata T, Yoshikawa M. 1986. A genetic determinant required for continuous reinfection of adjacent cells on large plasmid in S. flexneri 2a. Cell 46:551–555. doi:10.1016/0092-8674(86)90880-9 3524856

[53] Sansonetti PJ, Arondel J, Fontaine A, d’Hauteville H, Bernardini ML. 1991. OmpB (osmo-regulation) and icsA (cell-to-cell spread) mutants of Shigella flexneri: vaccine candidates and probes to study the pathogenesis of shigellosis. Vaccine (Auckl) 9:416–422. doi:10.1016/0264-410x(91)90128-s 1887672

[54] Desalegn G, Abrahamson C, Ross Turbyfill K, Pill-Pepe L, Bautista L, Tamilselvi CS, Dunn D, Kapoor N, Sullinger B, Herrera M, Oaks EV, Fairman J, Pasetti MF. 2025. A broad spectrum Shigella vaccine based on VirG_53–353_ multiepitope region produced in a cell-free system. NPJ Vaccines 10:6. doi:10.1038/s41541-025-01064-6 39805874 PMC11731012

[55] Leupold S, Büsing P, Mas PJ, Hart DJ, Scrima A. 2017. Structural insights into the architecture of the Shigella flexneri virulence factor IcsA/VirG and motifs involved in polar distribution and secretion. J Struct Biol 198:19–27. doi:10.1016/j.jsb.2017.03.003 28268178

[56] Shimanovich AA, Buskirk AD, Heine SJ, Blackwelder WC, Wahid R, Kotloff KL, Pasetti MF. 2017. Functional and antigen-specific serum antibody levels as correlates of protection against shigellosis in a controlled human challenge study. Clin Vaccine Immunol 24:1–13. doi:10.1128/CVI.00412-16 PMC5299116 27927680

[57] Goldberg MB, Theriot JA. 1995. Shigella flexneri surface protein IcsA is sufficient to direct actin-based motility. Proc Natl Acad Sci USA 92:6572–6576. doi:10.1073/pnas.92.14.6572 7604035 PMC41560

[58] Suzuki T, Miki H, Takenawa T, Sasakawa C. 1998. Neural Wiskott-Aldrich syndrome protein is implicated in the actin-based motility of Shigella flexneri. EMBO J 17:2767–2776. doi:10.1093/emboj/17.10.2767 9582270 PMC1170617

[59] Wang Y, Gong GH, Zhou W, Zhang B, Bao SY, Wei CX, Yue JJ, Zhang YF. 2014. Analysis on the interaction domain of VirG and apyrase by pull-down assay. Molecules 19:18090–18101. doi:10.3390/molecules191118090 25379645 PMC6271496

[60] Doyle MT, Tran ENH, Morona R. 2015. The passenger-associated transport repeat promotes virulence factor secretion efficiency and delineates a distinct autotransporter subtype. Mol Microbiol 97:315–329. doi:10.1111/mmi.13027 25869731

[61] Qin J, Hong Y, Morona R, Totsika M. 2022. Cysteine-dependent conformational heterogeneity of Shigella flexneri autotransporter IcsA and implications of its function. Microbiol Spectr 10:e0341022. doi:10.1128/spectrum.03410-22 36374106 PMC9769942

[62] Ogawa M, Yoshimori T, Suzuki T, Sagara H, Mizushima N, Sasakawa C. 2005. Escape of intracellular Shigella from autophagy. Science 307:727–731. doi:10.1126/science.1106036 15576571

[63] Jespersen MC, Peters B, Nielsen M, Marcatili P. 2017. BepiPred-2.0: improving sequence-based B-cell epitope prediction using conformational epitopes. Nucleic Acids Res 45:W24–W29. doi:10.1093/nar/gkx346 28472356 PMC5570230

[64] Kelley LA, Mezulis S, Yates CM, Wass MN, Sternberg MJE. 2015. The Phyre2 web portal for protein modeling, prediction and analysis. Nat Protoc 10:845–858. doi:10.1038/nprot.2015.053 25950237 PMC5298202

[65] Seo H, Garcia C, Ruan X, Duan Q, Sack DA, Zhang W. 2021. Preclinical characterization of immunogenicity and efficacy against diarrhea from MecVax, a multivalent enterotoxigenic E. coli vaccine candidate. Infect Immun 89:e0010621. doi:10.1128/IAI.00106-21 33875477 PMC8208516

[66] Upadhyay I, Li S, Ptacek G, Seo H, Sack DA, Zhang W. 2022. A polyvalent multiepitope protein cross-protects against Vibrio cholerae infection in rabbit colonization and passive protection models. Proc Natl Acad Sci USA 119:e2202938119. doi:10.1073/pnas.2202938119 36469767 PMC9897427

